# ATRI LIMS: a cloud-native open-source laboratory information management system for biomarker-driven Alzheimer’s disease clinical trials

**DOI:** 10.1093/jamiaopen/ooag198

**Published:** 2026-10-10

**Authors:** Gustavo A Jimenez-Maggiora, Stefania Bruschi, Sara Abdel-Latif, Paul S Aisen, Robert A Rissman

**Affiliations:** Epstein Family Alzheimer’s Therapeutic Research Institute, Keck School of Medicine of the University of Southern California, San Diego, CA 92121, United States; Epstein Family Alzheimer’s Therapeutic Research Institute, Keck School of Medicine of the University of Southern California, San Diego, CA 92121, United States; Epstein Family Alzheimer’s Therapeutic Research Institute, Keck School of Medicine of the University of Southern California, San Diego, CA 92121, United States; Epstein Family Alzheimer’s Therapeutic Research Institute, Keck School of Medicine of the University of Southern California, San Diego, CA 92121, United States; Epstein Family Alzheimer’s Therapeutic Research Institute, Keck School of Medicine of the University of Southern California, San Diego, CA 92121, United States

**Keywords:** data management, clinical trials, Alzheimer’s disease, biomedical informatics, biomarkers, laboratory information management systems

## Abstract

**Objectives:**

This article summarizes the architecture, design principles, and implementation strategy of the ATRI Laboratory Information Management System (LIMS), a cloud‑native, open‑source platform supporting biomarker‑driven Alzheimer’s disease (AD) clinical research. The system addresses challenges associated with managing large‑scale, multimodal biomarker, and biobanking data across multisite studies.

**Methods:**

The ATRI LIMS employs a Model–View–Controller architecture implemented in Django, with Single‑Page Application interfaces built in ReactJS. A hybrid database model integrates a normalized core schema with study‑specific extensions. Deployment on Amazon Web Services incorporates multifactor authentication, encryption, and a web application firewall. Regulatory support is provided through audit trails and electronic signatures. Development relied on collaboration between informatics engineers and laboratory personnel, with user acceptance testing guiding workflow and interface refinement.

**Results:**

As of February 2026, the ATRI LIMS supports seven clinical trials and observational studies and manages more than 1.2 million biospecimens. The platform synchronizes biospecimen metadata and assay outputs with clinical datasets, improves operational efficiency through automated workflows and quality control, and facilitates compliant sample sharing for secondary research. AI‑assisted development accelerated feature delivery while maintaining code quality.

**Discussion:**

The ATRI LIMS offers a scalable, adaptable informatics framework for biomarker‑intensive AD research. Its modular design, cloud‑native architecture, and comprehensive data management capabilities support accurate specimen tracking and coordinated data integration. An open-source release is planned to enable adoption and adaptation by other research programs.

**Conclusions:**

The ATRI LIMS enhances informatics infrastructure for modern AD research by enabling scalable, efficient, and compliant biomarker and biospecimen workflow management.

## Introduction

Alzheimer’s disease (AD) affects an estimated 6.9 million individuals in the United States and remains a major public health priority.^1^ Recent therapeutic advances, particularly the approval of disease-modifying monoclonal antibodies that target amyloid pathology, have substantially reshaped the design and implementation of AD clinical trials.^2–5^ Contemporary trials increasingly rely on biomarker-based diagnostic criteria, with cerebrospinal fluid (CSF) and blood-derived analytes, amyloid positron emission tomography (PET), and tau PET commonly used to confirm underlying AD pathophysiology and establish biological eligibility.^6^^,^^7^

The shift toward biomarker-driven clinical trials introduces significant laboratory, operational, and informatics complexity. Many trials now screen hundreds to thousands of candidates using plasma biomarkers with the goal of enriching cohorts for individuals most likely to exhibit AD pathology.^8–11^ Individuals with positive plasma screens may undergo confirmatory CSF analyses or amyloid PET imaging. Throughout a study, repeated biomarker assessments support target engagement analyses, disease trajectory characterization, and safety monitoring.

These workflows generate substantial data volumes. A single Phase III trial can produce approximately 10 000 to 50 000 biomarker data points across multiple assay platforms and time points. This scale underscores the need for robust biospecimen tracking, data management, and quality control systems capable of supporting multimodal biomarker pipelines.

The Epstein Family Alzheimer’s Therapeutic Research Institute (ATRI)^12^ in San Diego, part of the Keck School of Medicine of the University of Southern California, is dedicated to the development of effective treatments for AD and related dementias research. ATRI advances prevention, early detection, and therapeutic development through innovative clinical trials and methodological frameworks for evaluating candidate disease-modifying interventions. As the coordinating center for the NIH-funded Alzheimer’s Clinical Trials Consortium, ATRI provides scientific, operational, and regulatory oversight for multisite AD trials across the United States and supports large observational studies such as the AD Neuroimaging Initiative, which has played a central role in characterizing disease trajectories and accelerating biomarker discovery.

ATRI’s Neuroscience Translational Research Division^13^ Biomarker Core is a central component of the institute’s translational infrastructure. Serving as both a full-service experimental laboratory and a national biorepository, the Biomarker Core supports end-to-end biospecimen workflows that include receipt, standardized processing, analytical characterization, and long-term preservation for clinical trials and observational cohorts. As the primary biobanking resource for multiple federally funded AD initiatives, the Biomarker Core ensures rigorous quality control, regulatory and biosafety compliance, and secure stewardship of specimens to enable current and future investigations. Recent expansion increased the facility’s storage capacity to 248 ultra-low-temperature freezers, enabling storage of up to 11.57 million biospecimens and supporting large-scale biomarker discovery, validation, and translational research.

Biospecimen-intensive research of this scale requires advanced Laboratory Information Management Systems (LIMS) that support the complete biospecimen lifecycle, including chain-of-custody tracking, automated workflows, quality assurance procedures, instrument integration, biobanking operations, and regulatory compliance. Over the past decade, the Biomarker Core has implemented several commercial and open-source LIMS solutions to support its specialized biospecimen and data management needs. However, none of these systems provided the required flexibility, scalability, or interoperability to accommodate the growing complexity of AD research. Common limitations included rigid, workflow-centric architectures optimized for diagnostic or pharmaceutical quality control settings; high total cost of ownership for grant-funded programs; insufficient integration with research-grade laboratory instruments and analytic pipelines; vendor lock-in that imposed substantial migration and revalidation burdens; outdated user interfaces that hindered adoption and efficiency; and limited support for interinstitutional collaboration and controlled data and specimen sharing. These constraints motivated the development of a custom, research-grade LIMS specifically aligned with ATRI’s integrated biomarker, biobanking, and clinical research workflows.

ATRI’s Informatics and Data Management Section [14] has developed an integrated clinical research informatics platform that connects participant registry and remote prescreening systems with biomarker-informed precision screening and enrollment workflows, unifying laboratory, neuroimaging, and clinical data streams (Figure 1).^15^^,^^16^ During trial conduct, the platform supports multimodal data acquisition, standardized imaging workflows, centralized data management and safety monitoring operations, and coordinated operational oversight. It also interoperates with external systems responsible for critical trial operations, such as randomization and investigational product management, electronic clinical outcome assessments (eCOA), electronic patient-reported outcomes (ePRO), and neuroimaging infrastructure.^17^ These capabilities operate within a secure cloud-native architecture that provides the scalability, interoperability, and regulatory compliance required for large multisite clinical trials, supported by a unified data science environment and extensive administrative and regulatory tooling. Building on this informatics foundation and in response to the Biomarker Core’s need for a flexible, scalable, and interoperable LIMS, a custom solution was developed to integrate seamlessly with the existing platform and meet the specialized workflow, data management, and compliance requirements of biomarker-intensive AD research. The resulting ATRI LIMS supports the full biospecimen lifecycle across multiple concurrent studies and interfaces directly with clinical, imaging, and administrative systems to enable coordinated workflows and real-time data flow across the research enterprise.

**Figure 1.**
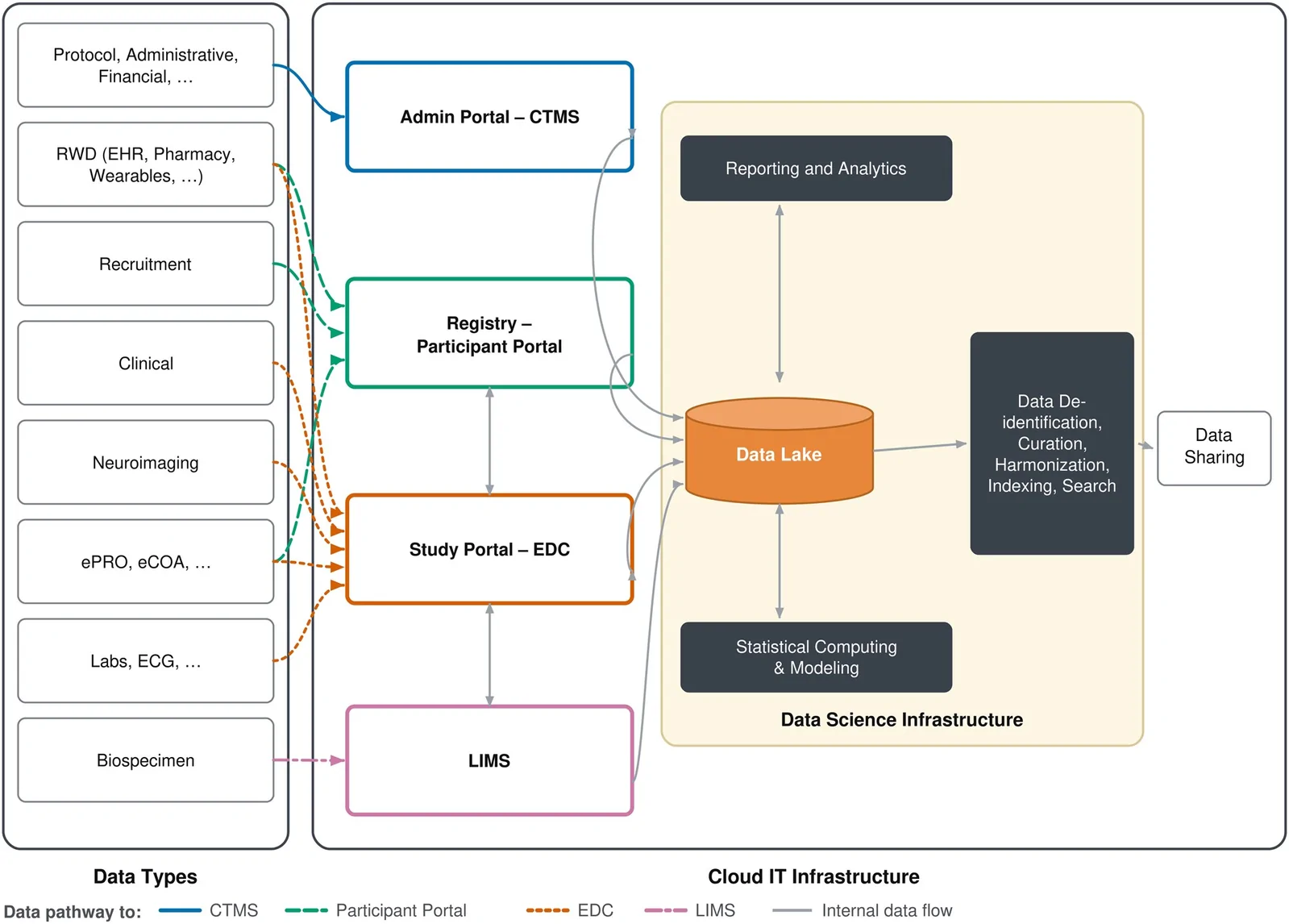
Overview of the ATRI clinical research informatics platform. This diagram illustrates the integrated informatics architecture that supports ATRI’s multisite clinical research operations. On the left, diverse data types—including protocol and administrative data, real-world data sources, recruitment information, clinical and neuroimaging data, electronic patient-reported outcomes (ePRO/eCOA), laboratory and physiological measurements, and biospecimens—enter the platform through domain-specific applications. These include the Administrative Portal and Clinical Trial Management System, the Participant Registry Portal, the EDC Study Portal, and the LIMS. All upstream systems feed into a centralized cloud-hosted Data Lake, which serves as the core of the Data Science Infrastructure. Within this environment, data undergo deidentification, curation, harmonization, indexing, and search optimization to support regulatory compliance and cross-study integration. The Data Lake supports downstream statistical computing, modeling, reporting, and analytics in real-time. Finally, curated datasets and analytic outputs can be shared through controlled data-sharing pathways, facilitating collaboration across research partners and supporting clinical and translational discovery efforts in AD and related disorders.

This article describes the architecture, design principles, and implementation strategy of the ATRI LIMS and demonstrates how the platform addresses the informatics challenges inherent in managing large-scale, multimodal biomarker, and biobanking data within a multisite clinical research enterprise. It further examines the system’s integration within ATRI’s research infrastructure, its contributions to operational efficiency and data governance, and its potential to accelerate clinical and translational research in AD and related neurodegenerative disorders.

The ATRI LIMS was designed to be portable and reusable beyond its originating institution, with a cloud-agnostic architecture and configurable, modular design that supports deployment across diverse research environments without requiring separate codebases. An open-source release is planned to facilitate broader adoption and community development, while the software is currently available from the corresponding author for evaluation. To support transparency and reproducibility, the Supplemental Materials provide a detailed inventory of the platform’s front-end, back-end, and cloud infrastructure components sufficient to guide independent implementation.

## Methods

### ATRI LIMS overview

The ATRI LIMS functions as a centralized informatics environment for managing clinical trials, observational studies, and biobanking initiatives conducted within the ATRI. The system provides a configurable framework that supports project-specific metadata schemas, workflow definitions, and access control models. Modular applications for specimen accessioning, shipment management, automated aliquoting, and facility metadata tracking enable the platform to accommodate heterogeneous scientific and operational requirements across studies (Figure 2).

**Figure 2.**
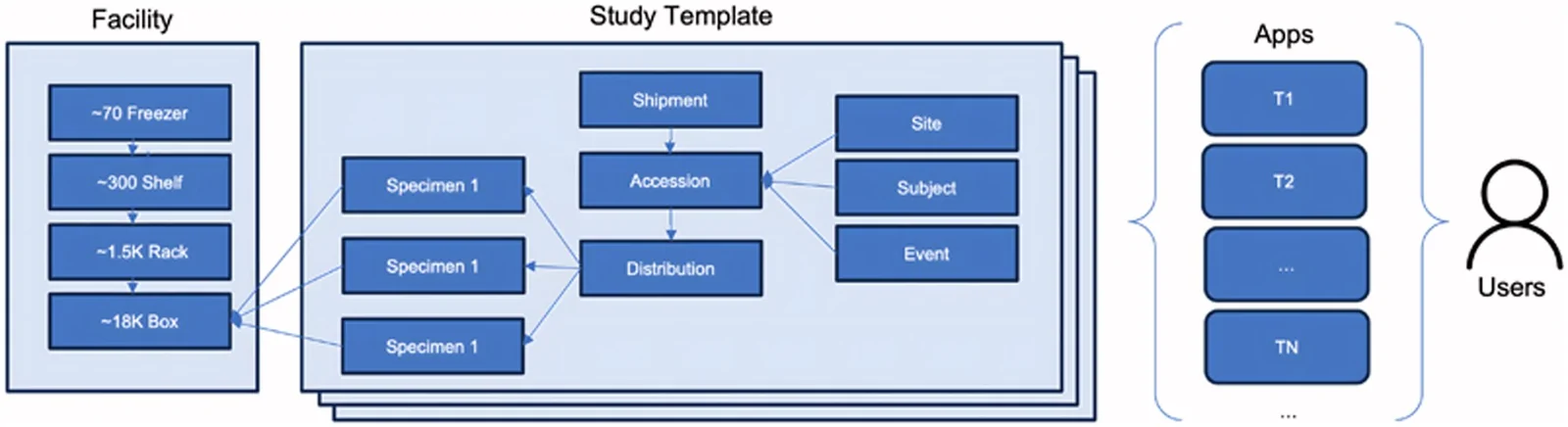
Conceptual architecture of the ATRI LIMS. The system links facility-level storage hierarchies (freezers, shelves, racks, and boxes) with study template entities that structure biospecimen collection, accessioning, distribution, and associated operational metadata such as sites, subjects, and events. Applications built on top of this model provide study-specific workflows, enabling users to manage biospecimen lifecycles consistently across multiple concurrent studies while maintaining alignment with the underlying facility and data structures.

The LIMS enables end-to-end management of the biospecimen lifecycle, beginning with specimen collection at participating clinical sites and extending through shipping, accessioning, storage, laboratory processing, analytical characterization, and long-term stewardship. Detailed representations of laboratory infrastructure, including freezer configurations, rack and box layouts, and complete inventory maps, support precise specimen localization and traceability. Embedded auditability, granular role-based permissions, and systemwide data integrity controls ensure compliance with regulatory and quality management requirements.

This modular and scalable design supports high-fidelity biospecimen management across ATRI’s research portfolio and provides a foundation for coordinated biospecimen-centric workflows in AD research. A complete listing of system requirements, including front‑end, back‑end, and cloud infrastructure services, is provided in the Supplemental Materials.

### System architecture

#### Model-view-controller architecture

The ATRI LIMS is implemented using a Model–View–Controller (MVC) architecture to support modularity, scalability, and maintainability. The Model layer defines the core data structures and business rules governing biospecimen entities, clinical and laboratory metadata, assay outputs, and role-based permissions. These elements are stored within a relational schema optimized for high-volume multimodal datasets and engineered to support robust auditability and regulatory compliance.

The View layer provides responsive, role-specific interfaces for laboratory technicians, data managers, and clinical researchers. Interfaces include interactive dashboards, workflow-driven electronic forms, and configuration-specific visualizations accessible across device types. The Controller layer mediates user interactions, implements application logic, enforces access control policies, validates inputs, and manages asynchronous operations such as batch uploads and automated reporting.

This separation of concerns enables distributed development, simplifies maintenance, and supports rapid adaptation to evolving research and operational requirements.

#### Django framework

The ATRI LIMS architecture is implemented using the Django web framework for Python, which provides a robust and maintainable foundation for data-intensive biomedical applications. Django’s structured routing, class-based views, templating system, and middleware support a clear MVC organization, while the Django REST framework enables rapid development of secure, standards-compliant web application programming interfaces (APIs) for interoperable data exchange. The framework’s stateless request–response model, modular application design, and native caching facilitate scalable deployment across distributed and cloud environments, including AWS Elastic Beanstalk. Horizontal scaling is supported through load balancing services, and integration with task queue systems such as AWS Batch enables asynchronous execution of computationally demanding workflows, including bulk ingestion, quality control pipelines, and scheduled reporting. Django’s Object‑Relational Mapper provides efficient and expressive access to relational databases, supporting complex queries and schema evolution while maintaining performance and code clarity.

#### Single‑page application interfaces

Each ATRI LIMS module is implemented as a Single-Page Application (SPA) to optimize usability and workflow efficiency. SPAs enable dynamic content updates within a persistent browser session, eliminating full page reloads and substantially improving responsiveness. This design allows users to move seamlessly between specimen tracking, data entry, and reporting interfaces during routine operations (Figure 3).

**Figure 3.**
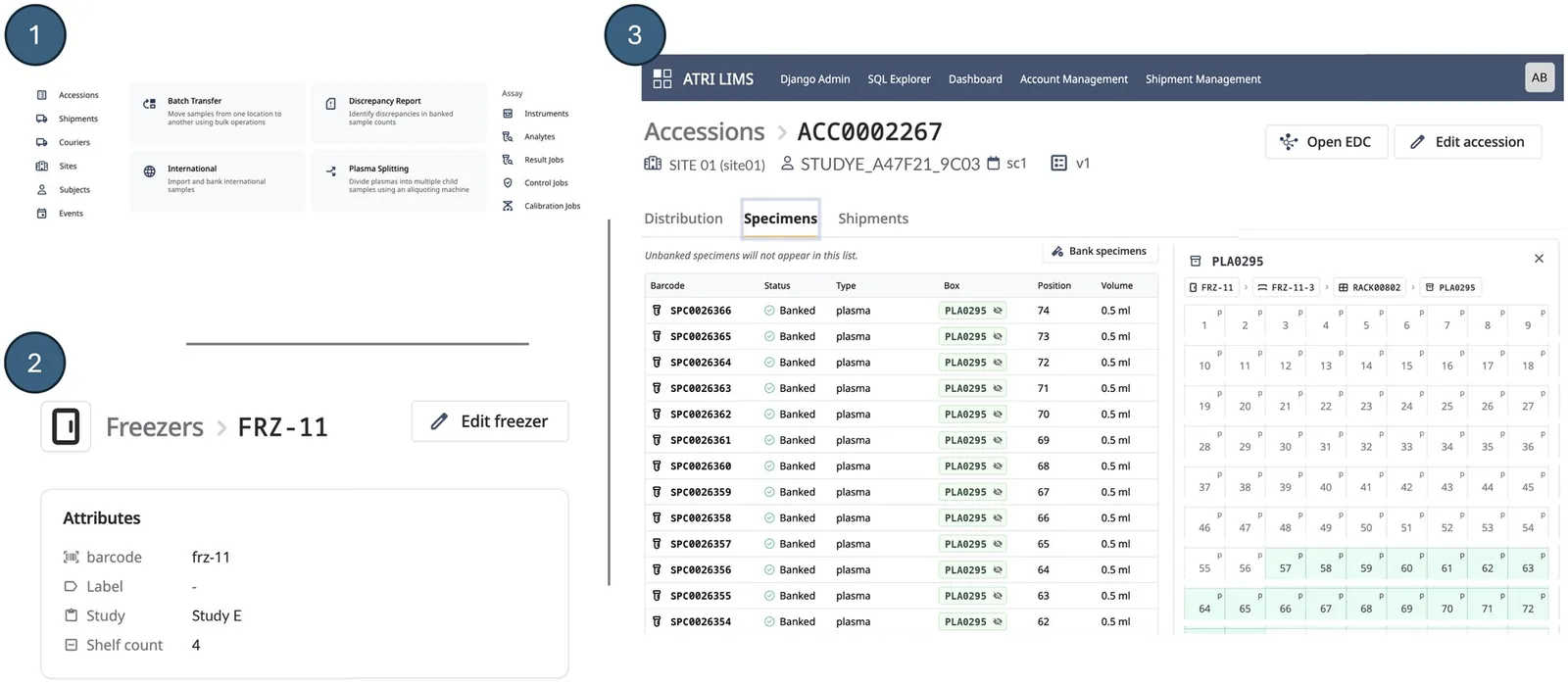
Representative ATRI LIMS user interface views. The interface provides (1) searchable and filterable inventory listings across facility and study contexts; (2) detailed entity-level views that display attributes for storage units such as freezers, shelves, and racks; and (3) accession-level dashboards that integrate specimen details, distribution history, and shipment records. Together, these views support real-time navigation of biospecimen inventories and streamline operational workflows across studies. Identifiers are masked to protect confidentiality.

User interfaces were prototyped in Figma and implemented in ReactJS, leveraging its component-based architecture, state management tools, real-time data synchronization capabilities, and integration with backend services. Application logic follows an object-oriented design in which abstract base classes encode shared functionality and project-specific subclasses implement study-specific features. This approach accelerates onboarding of new projects, standardizes feature deployment, and improves maintainability and testability across studies.

#### Database schema

The ATRI LIMS database schema uses a hybrid design that integrates a normalized core schema for foundational metadata with study-specific extensions that accommodate customized data structures and workflows. This approach preserves uniformity and interoperability across the platform while providing project-level flexibility to define metadata elements, specimen attributes, workflow steps, and analytical data fields tailored to individual study objectives, laboratory procedures, and regulatory requirements.

By avoiding a rigid monolithic schema, the system maintains conceptual coherence across studies while allowing controlled customization and evolution of data models. This design ensures that flexibility does not compromise system integrity, data quality, or long-term maintenance.

### Cloud‑native infrastructure

The ATRI LIMS is deployed as a cloud native system that leverages managed services provided by Amazon Web Services (AWS) (Figure 4). Core services include Elastic Beanstalk for automated provisioning, monitoring, and elastic scaling; Amazon S3 for durable, secure object storage; and Amazon Aurora PostgreSQL for high-performance relational data management with automated failover and continuous backup.

**Figure 4.**
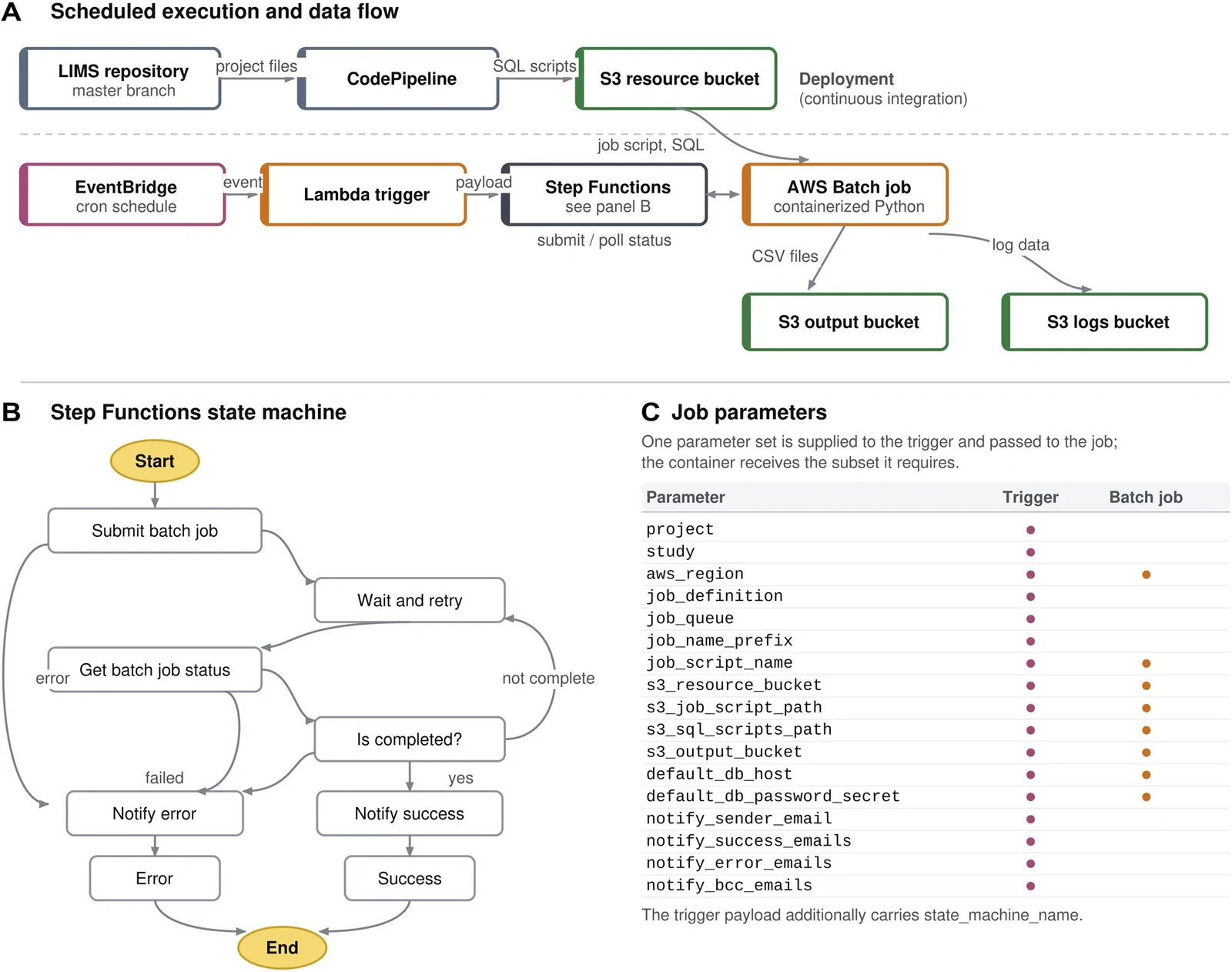
Serverless AWS architecture for automated execution of LIMS data-processing workflows. (A) Scheduled execution and data flow. SQL scripts and project files stored in the LIMS repository are deployed through CodePipeline to an Amazon S3 resource bucket. An Amazon EventBridge schedule triggers an AWS Lambda function, which initiates an AWS Step Functions workflow. The state machine submits and monitors a containerized Python job running in AWS Batch. Job outputs are written to an S3 output bucket, while execution logs are stored in a dedicated S3 logs bucket. (B) AWS Step Functions state machine. The workflow submits a batch job, periodically polls job status, retries while processing is incomplete, and routes execution to success or error notification paths based on the final job status. (C) Job parameterization model. A single configuration payload is supplied to the trigger and propagated through the workflow, with only the subset of parameters required for execution passed to the AWS Batch container, enabling reusable and configurable pipeline deployments across projects and studies.

Although optimized for AWS, the architecture remains portable and can be deployed on on‑premises infrastructure or alternative cloud providers, allowing adoption by institutions with diverse compliance requirements or IT constraints.

### Data security

The ATRI LIMS employs a comprehensive security architecture aligned with established best practices for biomedical informatics systems. Multifactor authentication is required for all users, and data is encrypted in transit and at rest using industry-standard cryptographic protocols. A web application firewall inspects incoming traffic to mitigate SQL injection, cross-site scripting, and related threats, while robust authentication and session management controls prevent unauthorized access and session hijacking. A multiregion disaster recovery strategy further ensures data durability and recoverability in the event of system failures or regional outages. Together, these measures safeguard the confidentiality, integrity, and availability of sensitive biomedical data.

### Regulatory compliance

The ATRI LIMS complies with 21 CFR Part 11 requirements for electronic records and electronic signatures. Immutable, time-stamped audit logs record all data modifications and associated contextual metadata, ensuring full traceability and accountability. Electronic signatures authenticate and authorize critical operations and approvals. These controls support regulatory compliance for clinical trials and other regulated biomedical research activities.

### Collaborative development process

The ATRI LIMS was developed through close collaboration between the Informatics Section and Biomarker Core teams. Informatics staff conducted workflow observations and iterative design reviews with laboratory personnel to ensure accurate alignment with real-world laboratory procedures, cognitive workflows, and usability needs. The multidisciplinary team included a senior software engineer, two software developers, a laboratory manager, and a laboratory data manager. Laboratory technicians contributed extensively through user acceptance testing, providing structured feedback that informed interface design, workflow refinement, and feature prioritization.

Key usability features include wireless barcode scanner integration, dynamic reporting and pick list generation, and automated reconciliation of specimen records with external systems. The platform also supports CDISC-compliant data import and export to ensure interoperability with downstream analytics and clinical trial data systems.

### AI‑enabled software development

AI‑assisted development tools, including GitHub Copilot and Copilot CLI, were integrated into the software engineering workflow to accelerate development and improve productivity. These tools assisted with routine code generation, refactoring, test creation, documentation, and contextual code recommendations. When combined with ATRI’s automated testing and validation frameworks,^18^ these tools significantly shortened development cycles and enabled faster implementation of new features. Their use also required specialized developer oversight to ensure correctness, maintainability, and compliance with software quality standards.

### Integration with clinical research systems

As a core component of ATRI’s clinical research informatics platform, the LIMS interoperates with ATRI’s Electronic Data Capture (EDC) systems,^16^ participant registries,^15^ and administrative platforms through standardized APIs and secure data exchange protocols. These integrations enable just-in-time synchronization of biospecimen metadata and assay results with clinical data streams, ensuring consistent alignment across operational systems. Connectivity with participant registries provides centralized tracking of participant information and specimen availability, whereas integration with administrative systems supports study oversight, regulatory compliance monitoring, and resource allocation. Interoperability with external vendor systems, including Randomization and Trial Supply Management (RTSM) platforms, further ensures coordinated execution of laboratory and clinical workflows.

Interactive dashboards provide real-time visibility into specimen flow, operational performance, and data quality across the clinical research network. Accrual dashboards display specimen and container registration volumes as calendar heat maps that can be filtered by study, specimen type, and time interval, enabling site and laboratory personnel to monitor collection activity, identify temporal trends, and detect deviations from expected accrual patterns. Distribution dashboards summarize banked specimens by type and disposition, including those distributed for analyses performed at the Biomarker Core and external analytical laboratories, with domestic and international shipments reported separately. These data are available as both graphical summaries and downloadable tabular reports. Facility dashboards characterize freezers and their internal storage components, including shelf, rack, and box utilization by study, supporting capacity management and long-term biorepository planning. Data quality dashboards monitor specimen accountability through discrepancy reports that compare the number of specimens banked for each accession with the number expected according to protocol-defined collection schedules, reporting signed differences to identify both missing and unexpected specimens requiring resolution. Instrument dashboards provide operational oversight of analytical platforms, including counts and timestamps of result-generation, quality control, and calibration activities, together with the associated versioned instrument configurations. Collectively, these dashboards support proactive issue identification, optimization of processing timelines, and data-driven decision-making, thereby enhancing trial conduct, participant safety, biomarker-enabled screening and enrollment workflows, intervention adherence, and the reliability of study outcomes (Figure 5).

## Results

### Data migration and system initialization

ATRI LIMS development was initiated in 2023, with initial efforts focused on implementing specimen inventory management as the foundational use case. The onboarding of specimen metadata and legacy datasets began in early 2024 and progressed in parallel with the development of new system features, ensuring uninterrupted operational continuity across active studies (Figure 6). Data migration involved extracting, transforming, and validating records from the prior LIMS solution to align with the new relational schema and metadata standards. Specimen-level records, assay results, and associated clinical metadata were mapped to study-specific configurations within the new architecture to preserve data integrity, maintain historical provenance, and ensure compliance with regulatory and quality management requirements.

**Figure 6.**
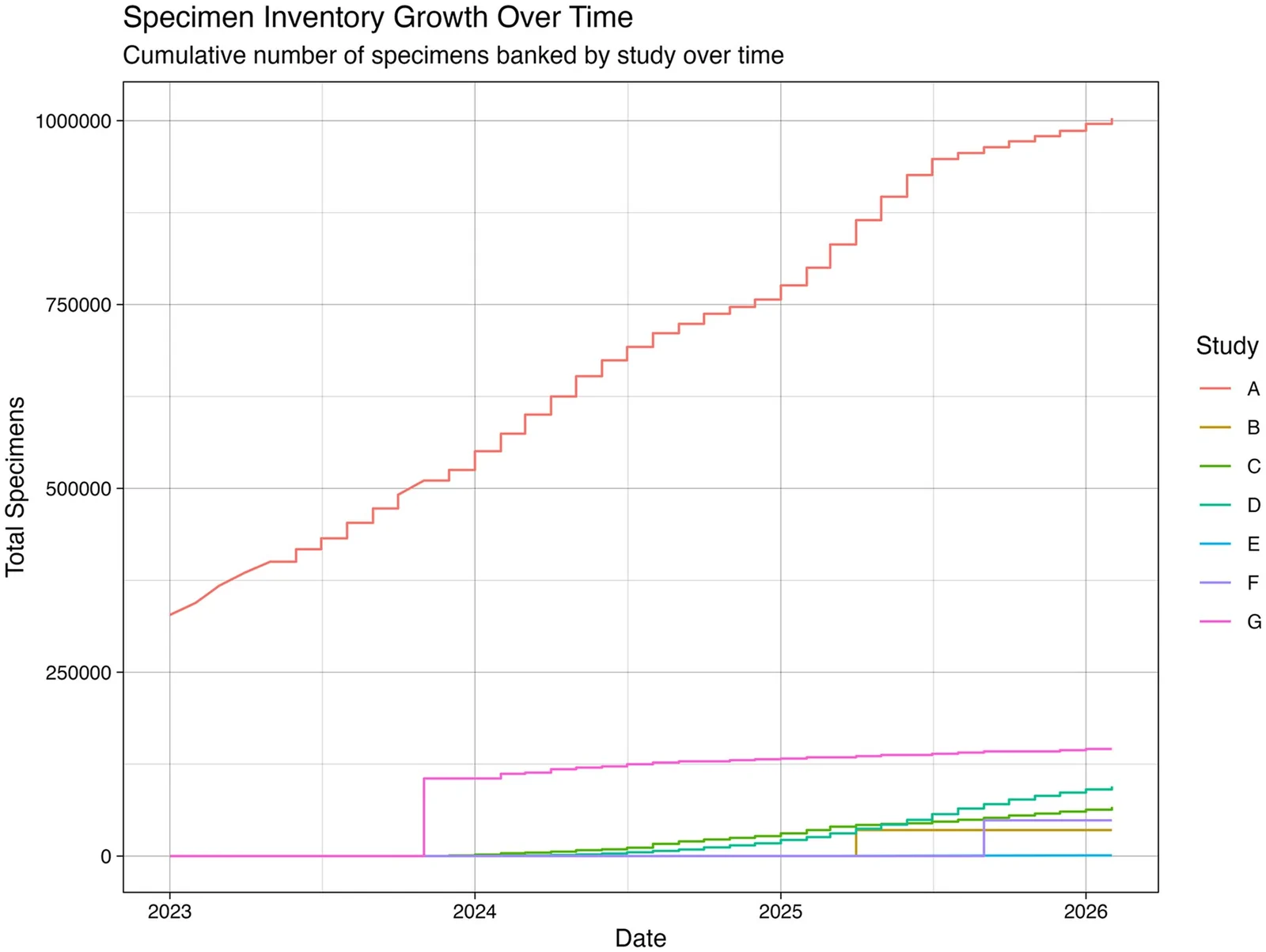
Specimen inventory growth over time (as of February 2026). Cumulative number of biospecimens banked over time across seven active ATRI studies. Each line represents the total number of specimens accrued by an individual study, with Study A contributing the largest volume and exhibiting continuous high throughput growth. Studies C-G show steady increases beginning in late 2023 to early 2024, reflecting staggered study initiations and varying collection intensities. The figure illustrates the expanding biospecimen inventory across the research portfolio and highlights study-specific differences in collection trajectories over the 2023-2026 period.

**Figure 5.**
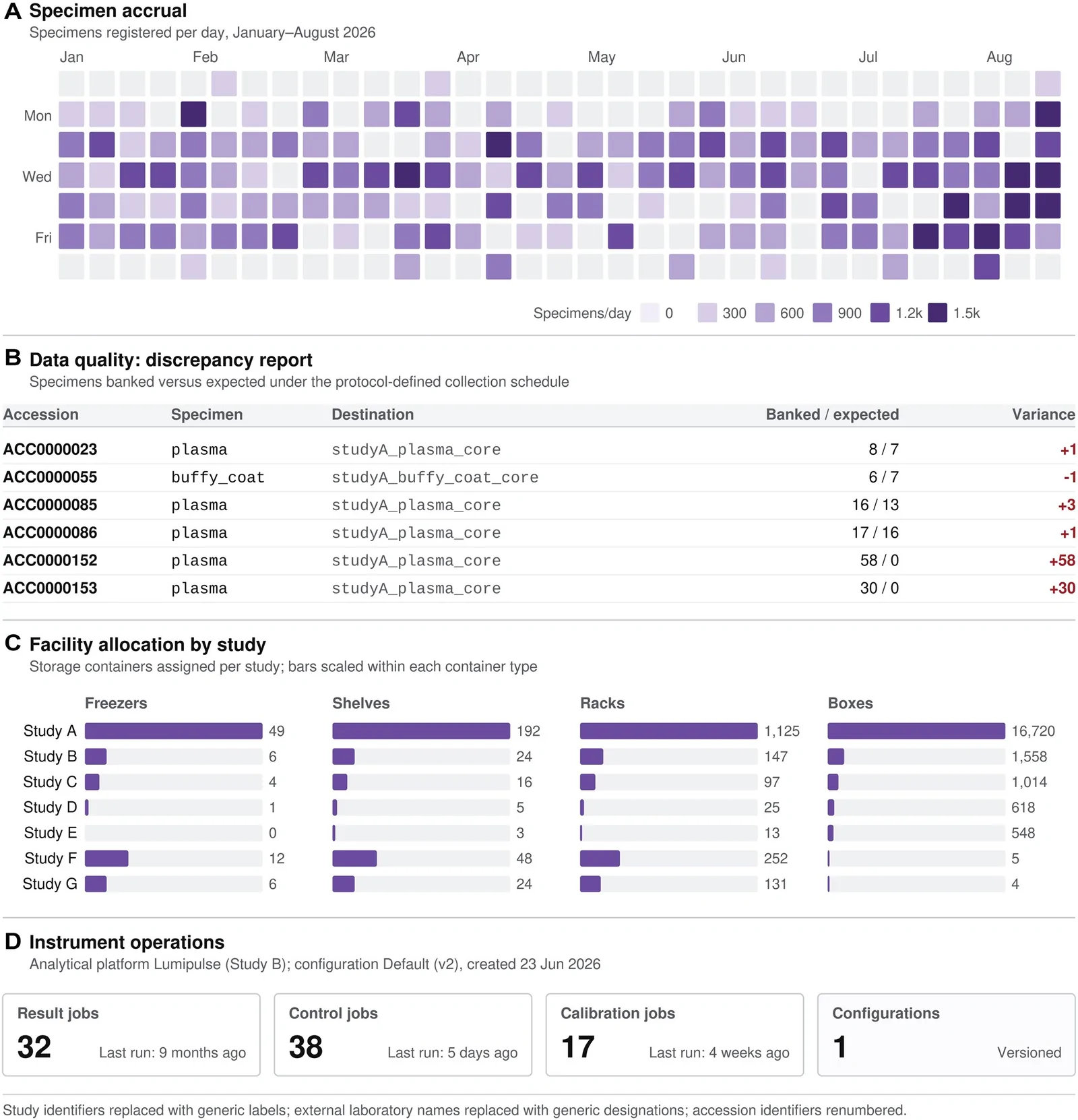
Representative ATRI LIMS operational and data quality dashboards (August 2026). (A) Daily specimen registration volume displayed as a calendar heat map. (B) Discrepancy dashboard comparing banked specimens with those expected according to the protocol-defined collection schedule for each accession; signed variance values identify both missing expected specimens and unexpected collected specimens. (C) Allocation of freezer, shelf, rack, and box capacity across seven active studies, with bar lengths scaled within each storage container type. (D) Instrument operations dashboard for a representative analytical platform, showing counts of result, quality control, and calibration jobs, together with the most recent execution times and the active instrument configuration version. Study identifiers, accession identifiers, and external laboratory names have been replaced with generic labels to preserve confidentiality. (Notes: panel A illustrates the structure of the accrual monitoring view; displayed values are representative and not derived from actual operational data. Storage containers supporting multiple studies are counted once for each associated study).

In addition to digital migration, the physical infrastructure of ATRI’s Biomarker Core was systematically represented in the LIMS. Freezers (71), racks (1528), boxes (19 254), box inventories, and analytical instruments were incorporated based on a comprehensive inventory curated by the laboratory manager. Inclusion of these facility-level metadata elements enabled precise specimen localization, improved storage capacity management, and facilitated integration of instrument metadata into downstream workflows such as calibration tracking and assay provenance.

Automated reconciliation scripts and validation utilities were applied to identify and resolve inconsistencies in migrated datasets, enforce business rules, and ensure internal coherence across study-specific configurations. Close coordination between informatics engineers and laboratory personnel supported the identification of essential metadata fields, refinement of domain-specific workflow requirements, and verification of study-level mappings. This coordinated strategy ensured continuous access to historical biospecimen data while enabling the system to expand in parallel with active project demands.

### System deployment across ATRI research programs

As of February 2026, the ATRI LIMS supports seven active projects spanning multisite clinical trials, observational cohorts, and biobanking initiatives (Table 1). Each project operates within a dedicated hybrid metadata schema, workflow specification, and access control model, enabling the platform to accommodate variations in study design, specimen types, laboratory methods, and regulatory requirements while maintaining interoperability and standardized governance across the shared system. The modular architecture of the LIMS has facilitated the rapid onboarding of new studies without disruption to ongoing research activities. Newly initiated projects were configured with study-specific workflows, specimen handling requirements, and analytical pipelines in a matter of weeks, demonstrating the system’s capacity to support flexible, parallel study operations.

**Table 1.** Study and specimen data summary (as of February 2026).^a^

Study^b^	Study data			Specimen data						
	Sites	Participants	Accessions	Blood	CSF	Buffy coat	Plasma	Serum	Urine	Other
A	115	14 563	35 865	–	7475	100 821	812 838	82 343	–	–
B	1	2255	2267	–	–	–	35 385	–	–	–
C	63	1349	2420	1345	–	–	65 518	–	–	–
D	58	1338	3849	–	1187	3999	89 544	–	–	–
E	14	62	190	–	–	–	–	–	942	–
F	1	1416	1424	–	–	–	48 569	–	–	–
G	–	–	–	2883	20 578	313	88 841	31 224	–	1769
Totals	252	20 983	46 015	4228	29 240	105 133	1 140 695	1 13 567	942	1769

a Cumulative number of participants, sites, accessions, and specimens across active studies.

b Study identifiers are masked to protect confidentiality.

### Biospecimen inventory growth and data integration

The ATRI LIMS currently manages more than 1.2 million biospecimens, including blood, CSF, plasma, serum, urine, and additional sample types collected across ATRI clinical trials and observational cohorts (Table 1). Each specimen is associated with rich metadata encompassing collection parameters, processing details, storage location, assay provenance, and longitudinal clinical context.

Dynamic specimen inventory maps, freezer rack abstractions, and real-time sample availability dashboards enable precise spatial tracking and prospective capacity planning for the Biomarker Core, which currently maintains storage infrastructure capable of housing up to 11.57 million biospecimens.

The system integrates laboratory-generated assay outputs with clinical and imaging data through standardized APIs, enabling real-time synchronization with EDC platforms and participant registry systems. This interoperability ensures consistent and coordinated data flow between laboratory and clinical operations teams, supporting timely eligibility assessments, disclosure of research results to participants, adaptive randomization decisions, and safety reviews in ongoing clinical trials.

A representative workflow illustrates how the ATRI LIMS links assay outputs to clinical data in practice. During screening for a biomarker-enabled trial, a participating site collects a plasma specimen at the screening visit and records the collection event in the EDC system. The specimen is shipped to the Biomarker Core, where barcode-driven accessioning in the LIMS reconciles the physical tube with the expected collection event, verifies specimen type, volume, and processing parameters against the protocol-specific configuration, and assigns a storage location. After analysis, instrument assay outputs—for example, plasma phosphorylated tau 217 (p-tau217) concentration and amyloid beta 42/40 (Aβ42/Aβ40) ratio—are imported in the LIMS and automatically linked to the specific aliquot analyzed, and protocol-defined quality control rules are applied. The results are then synchronized with the EDC system through a standardized API and attached to the participant’s screening record, where they are available to the clinical team for eligibility adjudication. Because the assay result, specimen chain of custody, and participant clinical record are linked through common identifiers, each biomarker value can be traced back to the specific aliquot, storage location, and processing history that produced it.

### Workflow efficiency and quality control enhancements

The configuration of laboratory workflows within the ATRI LIMS has resulted in measurable improvements in operational efficiency. Automated accessioning modules, wireless barcode scanner support, dynamic pick list generation, and laboratory supply logistics modules have reduced manual steps and minimized transcription errors.

Embedded quality control mechanisms validate data inputs, enforce protocol-specific rules, and generate automated alerts when outliers, missing data, or workflow deviations are detected. These alerts have been used by laboratory staff to identify issues in near real-time, improving the overall reliability of sample processing and data capture.

The standardized representation of facility metadata (eg, freezer configurations and rack box maps) also supports automated verification of storage locations, enabling rapid discrepancy resolution during audits, freezer migrations, or large-scale inventory reconciliations.

### Scalability and system performance

The cloud native deployment architecture has provided robust scalability. During periods of high assay throughput or intensive data entry activity, the system automatically scaled compute resources via AWS Elastic Beanstalk without downtime or performance degradation. Similarly, Amazon Aurora PostgreSQL has provided stable, high performance-transaction support, including during batch imports of laboratory data and integration events with EDC platforms.

Real-time and scheduled exports to ATRI’s clinical trial data lake allow near continuous availability of laboratory data for analytics, reporting, machine learning pipelines, and downstream statistical modeling. This integration has reduced data exchange latency from days to hours or minutes, depending on study configuration.

### Specimen and data sharing

The ATRI LIMS enables efficient selection, location, and controlled sharing of biospecimens and associated metadata for approved research through standardized request workflows and secure, role-based access. These processes are supported by audit trails and compliant data exchange protocols.

Aligned with ATRI’s commitment to open science,^19^ investigators receiving specimens are expected to return derived results for broader dissemination, enhancing transparency and maximizing specimen utility. As of February 2026, more than 2000 aliquots have been distributed for to external investigators for secondary research.

Interoperability with participant registries and EDC systems further integrates clinical, laboratory, and biospecimen data, facilitating coordinated data sharing and strengthening collaborative research efforts across the AD research community.

### Impact of AI‑enabled software development

The incorporation of AI‑assisted software development tools, including GitHub Copilot and Copilot CLI, substantially accelerated feature implementation and system optimization throughout the project period (Figure 7). These tools facilitated rapid generation of maintainable code, test suites, and documentation, and supported efficient refactoring and architectural improvements. Critically, all AI‑generated suggestions were subject to expert human review, ensuring that the gains in development velocity did not compromise code quality, regulatory alignment, or methodological rigor. When combined with ATRI’s automated testing and validation pipelines,^18^ this balanced approach to human–AI collaboration significantly shortened development cycles while maintaining the robustness, reproducibility, and reliability required for clinical research software.

**Figure 7.**
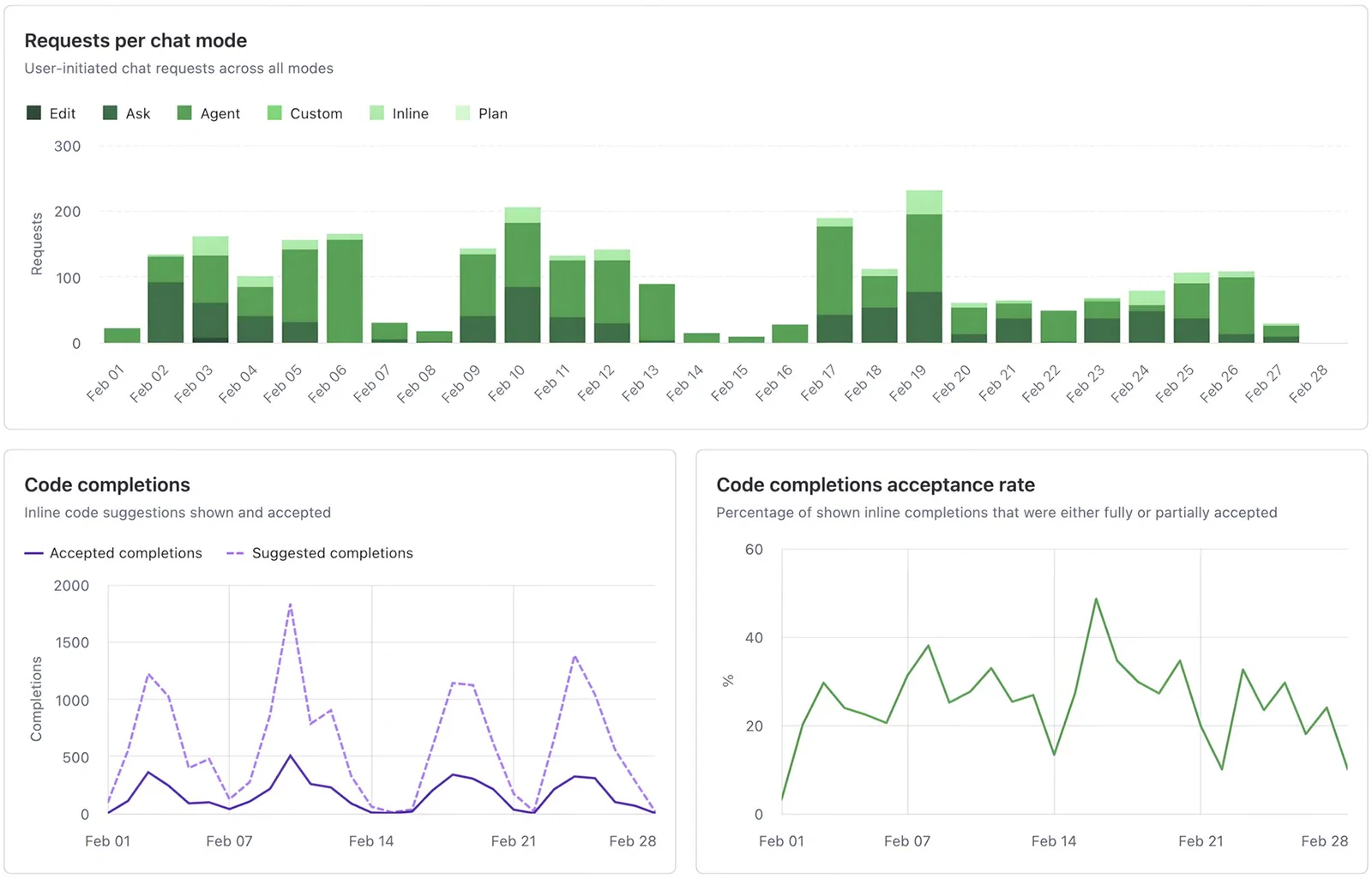
Utilization of GitHub copilot to enhance software development productivity and code quality within the ATRI informatics team (February 2026). This figure summarizes GitHub Copilot usage patterns and performance metrics across the ATRI Informatics development team. The top panel displays the volume of developer-initiated Copilot interactions by chat mode over time, including request types such as Ask, Agent, Edit, Custom, Inline, and Plan. The bottom left panel shows the number of inline code suggestions generated by Copilot and the subset accepted by developers, illustrating how AI-generated completions contribute to day-to-day coding tasks. The right bottom panel presents the acceptance rate of inline completions, representing the proportion of Copilot suggestions adopted by developers either fully or partially. Together, these metrics demonstrate how ATRI developers are leveraging Copilot to increase coding efficiency while retaining human oversight for quality control. The high and variable acceptance rates indicate active evaluation of AI-suggested code, supporting a workflow in which AI accelerates development, but final code quality is maintained through expert review and selective adoption of machine-generated contributions.

### Use beyond laboratory operations

Although the ATRI LIMS was developed primarily to support laboratory and study operations, its integration with ATRI’s EDC systems has extended its utility to a broad range of clinical, analytical, and research stakeholders. LIMS-derived reports embedded within the EDC allow clinical coordinators, monitors, and study teams to track specimen receipt, processing status, and assay completion in near real time, while discrepancy management workflows facilitate the rapid identification and resolution of collection, processing, and shipment issues throughout study conduct. By bringing specimen-level operational data into the clinical trial workflow, this integration has made specimen accountability a routine component of study oversight and monitoring. It also supports biomarker-informed screening and enrollment, eligibility adjudication, adaptive randomization, safety monitoring, and the assessment of study outcomes, thereby strengthening trial conduct, enhancing participant safety, improving intervention adherence, and reducing processing timelines.

The platform has also become an important resource for biostatisticians and data scientists. By integrating specimen metadata and assay provenance with clinical and imaging data in ATRI’s clinical trial data lake, the system enables analyses that incorporate laboratory variables such as processing parameters, storage duration, instrument assignment, and instrument configuration. The systematic capture of these data has extended the value of the LIMS beyond operational management, supporting more comprehensive and informative analyses of clinical and biomarker outcomes.

## Discussion

The ATRI LIMS provides an integrated informatics framework that addresses the growing operational and analytical demands of biospecimen-intensive AD clinical research. As AD trials increasingly incorporate multimodal biomarkers for diagnosis, eligibility assessment, participant stratification, and evaluation of treatment response, laboratory workflows and data management requirements have become substantially more complex. The findings presented here show that a modular, cloud-native, and interoperable LIMS can meet these demands at scale by supporting the complete biospecimen lifecycle and integrating effectively with clinical, imaging, and administrative workflows across multisite research programs.

A major strength of the ATRI LIMS is its ability to operationalize biomarker-driven clinical research protocols. These protocols require coordinated high-throughput plasma screening, confirmatory CSF or PET procedures, and repeated biomarker assessments throughout a study.^2–5^^,^^10^^,^^11^ Real-time synchronization of biospecimen metadata, assay results, and clinical information enables efficient eligibility adjudication, supports adaptive randomization strategies, and ensures timely access to data for safety monitoring and study oversight. The platform’s hybrid database architecture and flexible project-specific configurations allow it to accommodate diverse protocol designs, specimen types, assay platforms, and regulatory requirements while maintaining data integrity and interoperability.

The system also provides notable operational advantages. Cloud-native deployment ensures stable performance, elastic scalability, and rapid provisioning of resources during high-demand periods. Modular workflows, user-centered interfaces, dynamic reporting tools, and automated quality control mechanisms reduce manual burden and minimize error. Facility-level metadata integration enables precise specimen tracking, facilitates freezer migrations and audits, and simplifies biobank-scale inventory management.

Additionally, ATRI LIMS supports compliant and efficient biospecimen sharing for secondary research through controlled request workflows, comprehensive audit trails, and secure data exchange mechanisms that enforce robust governance and regulatory oversight. As of February 2026, more than 10 000 aliquots had been distributed to external investigators for secondary research, while over 100 000 aliquots had been released overall, including those supporting internal ancillary studies. By facilitating secure and traceable access to valuable biospecimens, the platform extends the scientific utility of participant contributions, accelerates biomarker and translational research, and advances ATRI’s commitment to open science and data sharing.

The system’s evolution has also benefited from the integration of AI-enabled software development tools, which have accelerated code optimization, test suite generation, and feature development without compromising quality. As AD biomarker technologies and clinical research methodologies continue to advance, such tools will play an increasingly important role in maintaining software adaptability, provided that appropriate oversight ensures reproducibility, regulatory compliance, and methodological rigor.

### Limitations

Despite its strengths, the system has inherent limitations. Dependence on cloud-based infrastructure may pose challenges for institutions with restrictive data governance frameworks or limited IT resources. The customizability that enables project-specific flexibility also increases complexity in system maintenance and may impose additional training burdens on new users. Sustained engagement with laboratory personnel remains essential to ensure that workflows remain aligned with evolving scientific, methodological, and regulatory requirements. Moreover, as AI-enabled tools become more deeply embedded in software development practices, the development team must remain attentive to emerging regulatory guidance and ensure that validation, documentation, and risk management practices evolve accordingly.^20^ These factors highlight the importance of ongoing system governance and continued collaboration between informatics, laboratory, and regulatory teams.

### Future directions

Looking forward, the ATRI LIMS is well-positioned to support the next generation of AD clinical research. Priority development efforts include expanding interoperability with additional data acquisition platforms, integrating real-time analytic capabilities, enhancing data visualization tools, and further automating quality control workflows. As new biomarker modalities and analytic approaches emerge, the system’s modular architecture will allow rapid incorporation of new instruments, assays, and computational pipelines, as well as future integration with laboratory automation and robotics systems. In the coming year, we intend to publish an open-source version of the ATRI LIMS to support broader adoption and collaborative development. In the interim, interested investigators may contact the corresponding author to request a copy of the software for evaluation purposes. More broadly, the platform provides a scalable and adaptable framework for biospecimen-centric informatics infrastructures applicable to other neurodegenerative disease programs and large research networks.

## Conclusions

The ATRI LIMS represents an advance in the informatics infrastructure required to support modern AD clinical research, particularly as studies increasingly rely on biomarker-driven trial designs. The platform enables scalable management of high-volume, multimodal biomarker and biospecimen workflows through a modular design, cloud-native deployment model, and comprehensive data management and compliance framework. These capabilities support precise tracking of specimens and analytical results across clinical trials, observational cohorts, and biobanking programs. Seamless interoperability with clinical, imaging, and administrative systems provides real-time cross-platform data flow, enhancing eligibility assessment, safety monitoring, and coordinated study operations in biomarker-based trials.

The rapid evolution of the system has been supported by the integration of AI-enabled software development tools, which accelerate feature implementation while maintaining code quality, reproducibility, and regulatory alignment. Continued collaboration among informatics, laboratory, operations, and regulatory teams will be essential to ensure the platform continues to match advances in biomarker science, laboratory methods, and emerging clinical trial designs.

As AD research advances toward increasingly data-intensive and biomarker-driven precision medicine paradigms, the ATRI LIMS is well-positioned to meet emerging scientific and operational demands. Its architectural principles and demonstrated performance offer a scalable framework for biospecimen-centric informatics infrastructures across other neurodegenerative disease programs and multisite research networks. Ongoing development will further enhance data quality, operational efficiency, and scientific rigor, ultimately accelerating the translation of biomarker-based discoveries into clinical impact.

## Supplementary Material

ooag198_Supplementary_Data

## Data Availability

No new data were generated or analyzed as part of this study.

## Declaration of the use of generative AI

During the preparation of this work, the authors used Microsoft Copilot (GPT-5) and Grammarly solely for language editing and copyediting to improve clarity, readability, and grammar. Following the use of these tools, the authors carefully reviewed and revised the manuscript as needed and take full responsibility for all content presented in this publication. Figures and images were relabeled to protect confidentiality and graphically refined using Anthropic Claude Cowork (Opus 5) to enhance presentation quality. No scientific content, analyses, results, interpretations, or conclusions were generated by these tools, and all scientific judgments remained the responsibility of the authors.
