# Supplementary material for: ATRI LIMS: a cloud-native open-source laboratory information management system for biomarker-driven Alzheimer’s disease clinical trials
Source: JAMIA Open. 2026 Oct 10;9(5):ooag198. doi: 10.1093/jamiaopen/ooag198 (PMC13653371; doi:10.1093/jamiaopen/ooag198)
Supplement: Supplementary file 1 [file ooag198_supplementary_data.docx]

**Supplemental Materials**

**Platform Requirements**

- Python v3.11.x
- Node v20.x

**Back-end Packages**

| Package | Version | Description | Repository URL |
| --- | --- | --- | --- |
| django | 4.2.16 | Backend web framework | [Repository](https://github.com/django/django) |
| psycopg2 | 2.9.10 | PostgreSQL database adapter for Python/Django | [Repository](https://github.com/psycopg/psycopg2) |
| django-simple-history | 3.7.0 | Tracks model changes over time for audit purposes | [Repository](https://github.com/jazzband/django-simple-history) |
| djangorestframework | 3.15.2 | Django library that aids in creating RESTful APIs | [Repository](https://github.com/encode/django-rest-framework) |
| dj-database-url | 2.3.0 | Allows the configuration of databases for Django via database URLs | [Repository](https://github.com/jazzband/dj-database-url) |
| requests | 2.32.3 | HTTP client for Python | [Repository](https://github.com/psf/requests) |
| pandas | 2.2.3 | Data analysis and manipulation library, used for spreadsheet ingestion and parsing | [Repository](https://github.com/pandas-dev/pandas) |
| django-storages | 1.14.4 | Adds cloud storage backend support for Django | [Repository](https://github.com/jschneier/django-storages) |
| boto3 | 1.35.51 | AWS SDK for Python | [Repository](https://github.com/boto/boto3) |
| django-json-widget | 2.0.1 | Django admin widget for editing JSON fields | [Repository](https://github.com/jmrivas86/django-json-widget) |
| django-admin-inline-paginator | 0.4.0 | Adds paginated inline relationship list components for the Django admin | [Repository](https://github.com/shinneider/django-admin-inline-paginator) |
| django-admin-searchable-dropdown | 1.2 | Adds widgets for autocomplete filters for the Django admin | [Repository](https://github.com/whoisashish/django-admin-searchable-dropdown) |
| django-admin-autocomplete-filter | 0.7.1 | Adds widgets for autocomplete filters for the Django admin | [Repository](https://github.com/farhan0581/django-admin-autocomplete-filter) |
| django-dynamic-raw-id | 4.3 | Improves the experience of using the “raw ID” feature for relationship fields in the Django admin | [Repository](https://github.com/lincolnloop/django-dynamic-raw-id) |
| django-import-export | 4.3.14 | Import and export database records via spreadsheet in the Django admin | [Repository](https://github.com/django-import-export/django-import-export) |
| django-admin-rangefilter | 0.13.2 | Adds widgets for filtering by numeric or temporal range for the Django admin | [Repository](https://github.com/silentsokolov/django-admin-rangefilter) |
| django-sql-explorer | 5.3.0 | Standalone Django app that allows users to execute SQL queries directly from a web interface | [Repository](https://github.com/groveco/django-sql-explorer) |
| jsonschema | 4.23.0 | Validates JSON data using schemas defined by the JSON Schema standard | [Repository](https://github.com/python-jsonschema/jsonschema) |
| typing_extensions | 4.12.2 | Backports of Python typing features for earlier Python versions | [Repository](https://github.com/python/typing_extensions) |

**Front-end Packages**

Packages versions adhere to the Semantic Versioning standard.

| Package | Version | Description | Registry Listing URL |
| --- | --- | --- | --- |
| @cypress/code-coverage | ^3.14.5 | Cypress code coverage support | [Registry listing](https://www.npmjs.com/package/@cypress/code-coverage) |
| @dnd-kit/core | ^6.1.0 | Core package for dnd-kit, which provides utilities for drag-and-drop UIs | [Registry listing](https://www.npmjs.com/package/@dnd-kit/core) |
| @dnd-kit/sortable | ^10.0.0 | Secondary package for dnd-kit, which provides utilities for drag-and-drop UIs | [Registry listing](https://www.npmjs.com/package/@dnd-kit/sortable) |
| @emotion/css | ^11.13.5 | CSS-in-JS styling library | [Registry listing](https://www.npmjs.com/package/@emotion/css) |
| @fortawesome/fontawesome-svg-core | ^6.7.2 | Font Awesome icon library - SVG core | [Registry listing](https://www.npmjs.com/package/@fortawesome/fontawesome-svg-core) |
| @fortawesome/free-regular-svg-icons | ^6.7.2 | Font Awesome icon library - regular icons | [Registry listing](https://www.npmjs.com/package/@fortawesome/free-regular-svg-icons) |
| @fortawesome/free-solid-svg-icons | ^6.7.2 | Font Awesome icon library - solid icons | [Registry listing](https://www.npmjs.com/package/@fortawesome/free-solid-svg-icons) |
| @fortawesome/react-fontawesome | ^0.2.2 | Font Awesome icon library - React bindings | [Registry listing](https://www.npmjs.com/package/@fortawesome/react-fontawesome) |
| @istanbuljs/nyc-config-typescript | ^1.0.2 | Config for TypeScript instrumentation for “nyc” coverage | [Registry listing](https://www.npmjs.com/package/@istanbuljs/nyc-config-typescript) |
| @nivo/calendar | ^0.87.0 | Calendar heatmap chart components | [Registry listing](https://www.npmjs.com/package/@nivo/calendar) |
| @nivo/core | ^0.87.0 | Core Nivo chart utilities | [Registry listing](https://www.npmjs.com/package/@nivo/core) |
| @nivo/pie | ^0.87.0 | Pie chart components | [Registry listing](https://www.npmjs.com/package/@nivo/pie) |
| @nivo/treemap | ^0.87.0 | Treemap chart components | [Registry listing](https://www.npmjs.com/package/@nivo/treemap) |
| @rjsf/core | ^6.0.1 | React JSON Schema forms | [Registry listing](https://www.npmjs.com/package/@rjsf/core) |
| @rjsf/utils | ^6.0.1 | JSON Schema form utilities | [Registry listing](https://www.npmjs.com/package/@rjsf/utils) |
| @rjsf/validator-ajv8 | ^6.0.1 | AJV v8 schema validator | [Registry listing](https://www.npmjs.com/package/@rjsf/validator-ajv8) |
| @tanstack/react-table | ^8.20.5 | Headless table library for React | [Registry listing](https://www.npmjs.com/package/@tanstack/react-table) |
| @testing-library/cypress | ^10.0.3 | Testing Library Cypress helpers | [Registry listing](https://www.npmjs.com/package/@testing-library/cypress) |
| @testing-library/jest-dom | ^5.17.0 | Jest DOM matchers | [Registry listing](https://www.npmjs.com/package/@testing-library/jest-dom) |
| @testing-library/react | ^14.3.1 | React testing utilities | [Registry listing](https://www.npmjs.com/package/@testing-library/react) |
| @testing-library/user-event | ^14.5.2 | User interaction simulation | [Registry listing](https://www.npmjs.com/package/@testing-library/user-event) |
| @total-typescript/ts-reset | ^0.6.1 | Safer TypeScript defaults | [Registry listing](https://www.npmjs.com/package/@total-typescript/ts-reset) |
| @types/cypress__code-coverage | ^3.10.2 | TypeScript types for Cypress coverage | [Registry listing](https://www.npmjs.com/package/@types/cypress__code-coverage) |
| @types/node | ^18.19.57 | Node.js type definitions | [Registry listing](https://www.npmjs.com/package/@types/node) |
| @types/react | ^18.3.23 | React type definitions | [Registry listing](https://www.npmjs.com/package/@types/react) |
| @types/react-dom | ^18.3.7 | React DOM type definitions | [Registry listing](https://www.npmjs.com/package/@types/react-dom) |
| @typescript-eslint/eslint-plugin | ^8.11.0 | TypeScript ESLint rules | [Registry listing](https://www.npmjs.com/package/@typescript-eslint/eslint-plugin) |
| @typescript-eslint/parser | ^8.11.0 | TypeScript ESLint parser | [Registry listing](https://www.npmjs.com/package/@typescript-eslint/parser) |
| @vitejs/plugin-react | ^4.3.3 | Vite React integration | [Registry listing](https://www.npmjs.com/package/@vitejs/plugin-react) |
| axios | ^1.7.7 | HTTP client library | [Registry listing](https://www.npmjs.com/package/axios) |
| bootstrap | ^5.3.3 | CSS framework | [Registry listing](https://www.npmjs.com/package/bootstrap) |
| cypress | ^14.5.0 | End-to-end testing framework | [Registry listing](https://www.npmjs.com/package/cypress) |
| eslint | ^8.57.1 | JavaScript linter which supports plugins | [Registry listing](https://www.npmjs.com/package/eslint) |
| eslint-config-prettier | ^9.1.0 | ESLint plugin for the prettier formatter | [Registry listing](https://www.npmjs.com/package/eslint-config-prettier) |
| eslint-import-resolver-typescript | ^3.6.3 | TypeScript import resolution | [Registry listing](https://www.npmjs.com/package/eslint-import-resolver-typescript) |
| eslint-plugin-cypress | ^3.6.0 | Cypress ESLint rules | [Registry listing](https://www.npmjs.com/package/eslint-plugin-cypress) |
| eslint-plugin-import | ^2.31.0 | ESLint import rules | [Registry listing](https://www.npmjs.com/package/eslint-plugin-import) |
| eslint-plugin-jsx-a11y | ^6.10.1 | JSX accessibility rules | [Registry listing](https://www.npmjs.com/package/eslint-plugin-jsx-a11y) |
| eslint-plugin-mocha | ^10.5.0 | Mocha ESLint rules | [Registry listing](https://www.npmjs.com/package/eslint-plugin-mocha) |
| eslint-plugin-react | ^7.37.1 | React ESLint rules | [Registry listing](https://www.npmjs.com/package/eslint-plugin-react) |
| eslint-plugin-react-hooks | ^4.6.2 | React Hooks lint rules | [Registry listing](https://www.npmjs.com/package/eslint-plugin-react-hooks) |
| eslint-plugin-react-refresh | ^0.4.13 | Fast Refresh lint rules | [Registry listing](https://www.npmjs.com/package/eslint-plugin-react-refresh) |
| eslint-plugin-tsdoc | ^0.3.0 | TSDoc lint rules | [Registry listing](https://www.npmjs.com/package/eslint-plugin-tsdoc) |
| fuse.js | ^7.0.0 | Fuzzy search library | [Registry listing](https://www.npmjs.com/package/fuse.js) |
| prettier | ^3.3.3 | Code formatter | [Registry listing](https://www.npmjs.com/package/prettier) |
| react | ^18.3.1 | Frontend component framework | [Registry listing](https://www.npmjs.com/package/react) |
| react-dom | ^18.3.1 | React sub-package for adding support for creating React HTML components | [Registry listing](https://www.npmjs.com/package/react-dom) |
| react-bootstrap | ^2.10.5 | Adds React components that correspond to Bootstrap components | [Registry listing](https://www.npmjs.com/package/react-bootstrap) |
| react-hook-form | ^7.53.1 | Form state management | [Registry listing](https://www.npmjs.com/package/react-hook-form) |
| react-router | ^6.27.0 | Client-side routing | [Registry listing](https://www.npmjs.com/package/react-router) |
| react-router-dom | ^6.27.0 | DOM bindings for React Router | [Registry listing](https://www.npmjs.com/package/react-router-dom) |
| react-select | ^5.8.1 | Select input component with dropdown/autocomplete | [Registry listing](https://www.npmjs.com/package/react-select) |
| react-toastify | ^10.0.6 | Toast notifications | [Registry listing](https://www.npmjs.com/package/react-toastify) |
| sass | ~1.63.6 | CSS preprocessor | [Registry listing](https://www.npmjs.com/package/sass) |
| typescript | ^5.8.3 | The official library for the TypeScript programming language | [Registry listing](https://www.npmjs.com/package/typescript) |
| usehooks-ts | ^3.1.0 | Reusable React utility hooks | [Registry listing](https://www.npmjs.com/package/usehooks-ts) |
| vite | ^5.4.9 | Frontend source file build tool | [Registry listing](https://www.npmjs.com/package/vite) |
| vite-plugin-checker | ^0.8.0 | Error/warning indicator for Vite | [Registry listing](https://www.npmjs.com/package/vite-plugin-checker) |
| vite-plugin-istanbul | 6.0.0 | Vite code coverage plugin | [Registry listing](https://www.npmjs.com/package/vite-plugin-istanbul) |
| vite-tsconfig-paths | ^5.0.1 | Auto-configuration tool for configuring Vite with TypeScript | [Registry listing](https://www.npmjs.com/package/vite-tsconfig-paths) |
| xlsx | ^0.18.5 | Spreadsheet file parser | [Registry listing](https://www.npmjs.com/package/xlsx) |

**Cloud Infrastructure Services**

| Name | Description | Link |
| --- | --- | --- |
| Google Analytics | Tracks and reports website traffic | <https://analytics.google.com/analytics/web/> |
| AWS | Elastic Beanstalk | |
|  | S3 bucket | |
|  | CloudWatch | |
|  | RDS / Aurora | |
|  | Simple Email Service | |
|  | Code Pipeline | |
|  | Cloud Formation | |
| GitHub Actions | Testing Framework - CI - TDD | [GitHub Actions documentation - GitHub Docs](https://docs.github.com/en/actions) |
| Coveralls | Testing Framework - TDD Coverage | [Coveralls.io - Test Coverage History and Statistics](https://coveralls.io/) |
